# Effect of Power Ultrasound Treatment on Free and Glycosidically-Bound Volatile Compounds and the Sensorial Profile of Red Wines

**DOI:** 10.3390/molecules26041193

**Published:** 2021-02-23

**Authors:** Rodrigo Oliver Simancas, María Consuelo Díaz-Maroto, María Elena Alañón Pardo, Paula Pérez Porras, Ana Belén Bautista-Ortín, Encarna Gómez-Plaza, María Soledad Pérez-Coello

**Affiliations:** 1 Area of Food Technology, Faculty of Chemical Sciences and Technologies, Regional Institute for Applied Scientific Research (IRICA), University of Castilla-La Mancha, Avda. Camilo José Cela 10, 13071 Ciudad Real, Spain; rodrigo.oliver@uclm.es (R.O.S.); mariaconsuelo.diaz@uclm.es (M.C.D.-M.); soledad.perez@uclm.es (M.S.P.-C.); 2 Area of Food Technology, Higher Technical School of Agronomic Engineering, University of Castilla-La Mancha, Ronda de Calatrava 7, 13071 Ciudad Real, Spain; mariaelena.alanon@uclm.es; 3 Department of Food Science and Technology, Faculty of Veterinary Sciences, University of Murcia, 30071 Murcia, Spain; paula.perez2@um.es (P.P.P.); anabel@um.es (A.B.B.-O.)

**Keywords:** ultrasounds, wine, volatile compounds, aroma

## Abstract

This study presents the effect of the application of high-power ultrasound to crushed grapes, at a winery-scale, on the content of varietal volatile compounds (free and glycosidically-bound) in musts and on the overall aroma of wines. Two different frequencies (20 kHz and 28 kHz) were tested and the combination of grape sonication and different maceration times on wine aroma was also evaluated. The volatile compounds were isolated by solid phase extraction and analyzed by gas chromatography-mass spectrometry, carrying out a sensory evaluation of wines by quantitative descriptive analysis. Sonication produced an increase in the concentration of free varietal compounds such as C_6_ alcohols, terpenes and norisoprenoids in musts and also in wines made by 48 h of skin maceration, being less efficient in the extraction of the bound fraction. Fermentation compounds were also positively affected by ultrasound treatment, although this effect was variable depending on the frequency used, the maceration time and the type of compound. All the wines made from sonicated grapes had better scores in the evaluated olfactory attributes with respect to the control wines. Our results indicate that sonication could produce an increase in the content of some volatile compounds of sensory relevance, obtaining wines with an aroma quality similar or higher than those elaborated with longer maceration times.

## 1. Introduction

In recent years, the oenological industry has undergone a great evolution, introducing innovative technologies in the wine-making industry to optimize and make processes profitable, as well as to achieve higher product quality but also increase its diversification. In any case, acceptance by the consumer is essential, being the aroma one of the factors that most influence the sensory evaluation of wine.

The final aroma of the wine involves a high number of volatile compounds which are formed by diverse biological, biochemical and technological processes, such as grape metabolism, pre-fermentation processes, metabolism of microorganisms (alcoholic and malolactic fermentation) and chemical and enzymatic reactions that occur during the aging of wine.

Grape-derived compounds (primary or varietal aroma) play an important role in the character of a wine, as they usually present pleasant aromas related to the grape variety. Many of these compounds are found in the grape skin, in many cases in the form of precursors (glycosides, fatty acids, phenolic acids, amino acids, S-cysteine conjugates) that release odoriferous molecules through enzymatic or chemical hydrolysis [1].

Glycosidic precursors have been the most studied since many of the compounds of great sensory relevance in wines, such as terpenes, C_13_-norisoprenoids and benzene compounds, can be present in grapes in both free odor-active forms or linked to sugar molecules (glycoconjugates) [2]. Different practices have been used to promote the hydrolysis of glycosidically linked compounds: using strains of Saccharomyces and other yeasts with glycosidase capacity, increasing contact with the grape skins, or by adding exogenous glycosidic enzymes at the end of alcoholic fermentation [3,4,5].

Grape maceration is one of the process presenting a notable influence in the organoleptic quality of wines. This process involves the contact between the must and the solid parts of the grape, getting the extraction and dissolution of substances present in the grape skins, such as phenolic compounds, volatile compounds and their precursors. In the case of red wine, grape maceration continues for several days after the start of the alcoholic fermentation, obtaining an increased in the extracted compounds thanks to the ethanol formed.

Different techniques have been developed to promote the migration of compounds by breaking the cell tissue of the grape (pectinolytic enzymes, cryomaceration, thermovinification, flash release systems…) [6,7]. Many of them have been applied in red wine vinification to achieve an increase in the extraction of phenolic compounds shortening the maceration times. However, organoleptic changes due to the extraction of grape volatile compounds, the release of volatile compounds from their precursors, or the possible formation of off-flavors must be considered. Cryo-maceration favors the extraction of varietal aromas such as monoterpenes [8,9]. Thermovinification causes thermal degradation of grape precursors and an increase in esters, acids and fatty acids, as well as off-flavors production [10]. But also, the application of relatively strong temperatures causes a decrease in terpenes, probably by the denaturation of endogenous enzymes [11].

In recent years, unconventional techniques such as microwave processing and electric pulses have been proposed to promote the extraction of phenolic and volatile compounds from grapes and other fruits, reduce extraction times and increase the yield and quality of products [12,13].

The International Organization of Vine and Wine has recently approved the use of a new technology based on sonication to increase extraction during winemaking [14]. The ultrasound technique is based on the use of mechanical sound waves with frequencies between 20 kHz and 10 MHz. Depending on the frequency and the energy applied, a difference is made between high-frequency ultrasound (100 kHz–1 MHz) and high-power ultrasound (20–100 kHz) [15,16].

The high-power ultrasound (HPU) technique is an innovative technological process used in the food industry with different applications (stabilization, degassing, homogenization, extraction) [17,18,19]. Specifically, in the wine industry it has been used for microbiological stabilization of wine [19,20,21], to accelerate the aging process of wine [22,23], to promote the extraction of phenolic compounds during the skin maceration process [12,24,25] and to reduce the amount of alcohol in red wine [26].

However, the effects of sonication in wine and other foods have yet to be resolved. The conditions created in the sonication process can lead to the breakdown or degradation of solutes [27]. The use of ultrasound (US) was responsible for the appearance of secondary degradation products of free radical-induced lipid oxidation in treated milk [28] and in sonicated olive oil, given rise to off-flavors formation [29].

Regarding wine, US has been shown to influence the electrical conductivity of red wine, activate the formation of free radicals and cause changes in the aroma and sensory characteristics of wines [22,30].

The effects of the use of US in winemaking on wine aroma may differ depending on the time, frequency and treatment conditions used [31]. Some authors describe an enhancing effect of the aroma of the US treated wines and greater complexity [32], while others did not find significant differences in the content of most volatile compounds in wines from sonicated grapes compared to control wine [24].

It should also be noted that most studies have been conducted with ultrasonic baths or probes at laboratory scale. With the aim of studying the applicability of US at winery scale, a large-scale system, capable of working at different frequencies, was tested. Its application to crushed grapes increased tannin and anthocyanin concentration in wines, the results being frequency-dependent [25], however the effect on must and wine volatile compounds was not studied. On the other hand, just one study has been found regarding the use of US to the extraction of grape precursors, specifically in the extraction of thiols precursors of Sauvignon Blanc grapes [33]. Therefore, the aim of this study was to evaluate for the first time the effect of the ultrasound treatment of crushed grapes, at winery scale and at two different frequencies (20 and 28 kHz), on the free and glycosidically-bound volatile compounds and aroma profile of musts and their resulting red wines.

## 2. Results and Discussion

### 2.1. Effect of Ultrasounds Treatment on Free and Glycosidically Bound Volatile Compounds of Musts

**Figure 1:**
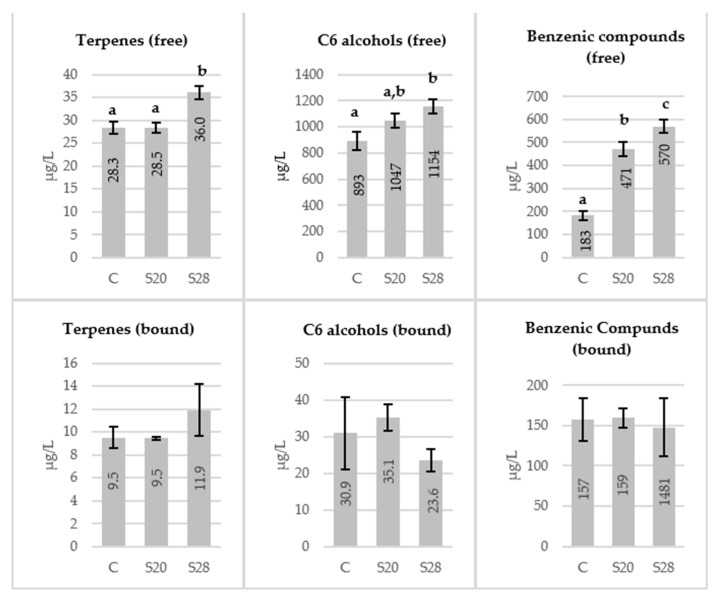
Mean concentrations (μg/L) of the main varietal compounds (free and bound fraction) regarding control and sonicated musts. ^a,b,c^ Different superscripts denoted significant differences according to the Student-Newman-Keuls test at *p* ˂ 0.05. C: control must; S20: must from grape sonicated at 20 kHz; S28: must from grape sonicated at 28 kHz.

The must samples were collected after the grape crushing and sonication treatment. They were centrifuged and the analysis of the free and bound fraction of the aroma was carried out according to the methodology described. Figure 1 shows the total quantities of the main groups of varietal compounds analyzed in the musts. Musts from sonicated grapes at 28 kHz contained significantly higher concentrations of free terpenes, C6-alcohols and benzene compounds compared to the control must from untreated grapes. While must from grapes sonicated at 20 kHz showed significant differences only in the case of free benzene compounds (Figure 1).

The varietal compounds found in the highest concentration in each must were C_6_-alcohols, with 1-hexanol as the majority compound (Supplementary Materials Table S1). These compounds are formed by the degradation of grape cell membrane lipids, favored by exposure to oxygen during the pre-fermentation processes [1]. Therefore, those processes which imply a greater intensity of the skin degradation and/or longer maceration will increase their amount in the grape must and consequently in the wine [5].

Benzene compounds were quantitatively important as well, with benzyl alcohol and 2-phenylethanol being the majority in all samples. These compounds were the most affected by the ultrasound treatment since their quantity increased more than 30% with sonication. In their free form, they come from the phenolic acids of the grape. However, these compounds are usually found mostly in their bound form, so their release through glycosidic enzymes or intense macerations is very effective [2].

Terpenes, located mainly in the grape skins, were quantitatively less important and the least affected by sonication. Three terpenes were identified in the musts: linalool, 1,4-terpineol and *trans*-geraniol, although only *trans*-geraniol presented significantly higher concentrations in the sonicated must at 28 kHz (Table S1, in the Supplementary Materials). On the other hand, higher amounts of terpenes and benzene compounds were obtained at 28 kHz, indicating a greater effect of ultrasound extraction at higher frequencies [34].

Regarding the glycosidically bound fraction, no significant differences were found in the different groups of varietal compounds due to the sonication treatment (Figure 1). Benzene compounds presented the highest amount of glycosidically bound fraction, while this is a minority fraction in the case of the C_6_ alcohols, as has been described in other grape varieties [3].

Comuzo et al. [13] observed in musts from grapes treated with pulsed electric field processing, an increase in free geraniol, while linalool was not affected. However, the concentration of all terpenes and norisoprenoids increased in the glycosidically bound fraction.

Studies carried out on the use of US in grape maceration and its effect on the volatile fraction are focused on wines, so there are few references on its effect on musts. The only work in which the effect of US treatment on aroma precursors in musts has been studied is that carried out by Roman et al. [33]. These authors did not observe a positive effect on the extraction of thiols precursors in Sauvignon Blanc grapes treated with an ultrasound probe at 20 kHz. However, an increase of free thiols in the same grape variety after grape sonication has been demonstrated [35]. This research justifies this behavior due to the breakage of the precursors following the US treatment, since the breakage of other molecules in sonicated samples has been previously described [36].

Sonication causes the formation of cavitation bubbles that can collapse violently causing a release of energy, raising the temperature and pressure. These bubbles can chemically interact with molecules or collapse near a solid surface, causing damage to cell structures and increasing the permeability of cell membranes or changing their selectivity [37]. In this way, the increase observed in the concentrations of free volatile compounds in musts from sonicated grapes could be due both to a release of their precursors and to a greater extraction from the solid parts of the grapes. This effect is clearly seen in the case of benzene compounds, where the bound fraction was similar in control musts and those treated with ultrasound, while the free fraction considerably increases their concentration in sonicated musts (Figure 1).

### 2.2. Effect of Ultrasounds Treatment on Free and Glycosidically Bound Volatile Compounds of Control Wines and Those Made from Sonicated Grapes

The control and sonicated musts were vinified, with two different maceration times being tested, 48 and 72 h. Moreover, the volatile compounds of a control red wine with 7 days of maceration were also compared. Table 1 and Table 2 show the concentration of main chemical groups of varietal compounds (free and bound fraction) identified in the wines analyzed after bottling.

C_6_-alcohols can continue to undergo modifications during the winemaking process, especially during the skin maceration stage due to the release of lipoxygenase enzymes from the grape skin cells. As in grape musts, 1-hexanol was the majority C_6_-alcohol in all wines.

The effect of ultrasound treatment was observed mainly in wines obtained from grapes sonicated at 20 and 28 kHz with 48 h of skin maceration, which had higher amounts of hexanol and *trans*-2-hexen-1-ol. In previous studies, it was observed that the effect of Monastrell grape sonication at 28 kHz on this group of compounds was not significant [24,26]. However, Ruiz-Rodriguez et al. [32] reported an increase between 20% and 60% in the amounts of 1-hexanol and *cis*-3-hexen-1-ol when the same frequency was applied during the fermentation of Syrah wines.

C_6_-alcohols have green and herbaceous notes and can contribute freshness to young wines, especially white wines. However, in high quantities they can generate unpleasant aromas, so an increase in their concentration is undesirable for the sensory quality of wines. In our case, the amounts found in all the wines were below their olfactory perception thresholds [1].

The bound fraction of C_6_ compounds was very small and few changes were found due to grape sonication (Table 2). Terpenes and their derivatives have a great sensory impact in wines from aromatic varieties such as Muscat, due to their low odor thresholds and pleasant aromas (floral or fruity), but they are less relevant in the aroma of wines from other grape varieties, especially in the case of red wines [38]. Although they come from the grape, many of them undergo modifications during fermentation, mainly by the liberation of its precursors due to the possible glycosidic activity of yeasts [4,39]. Hydration or oxidation reactions can also affect terpenes, giving rise to compounds with lower aromatic potential. This is the case of hydroxycitronellol and hydroxylinalool formed from linalool [40].

Linalool, 1-4-terpineol, citronellol and geranyl acetate were the most abundant terpenes in the tested wines, but only linalool exceeded its odour threshold in all of them (Table 1). In general, sonication did not enhance the extraction of terpenes. No significant differences were observed in most of these compounds in wines made from sonicated grapes, but those from grapes sonicated at 28 kHz with 72 h of skin maceration presented lower amounts of linalool, 4-terpineol and citronellol.

Nor-isoprenoids are a diverse group of compounds derived from the oxidative breakdown of grape carotenoids. The β-damascenone (floral, sweet and honey like aroma) presents a low perception threshold (0.05 μg/L) [41], which was exceeded in all analyzed wines. This compound has been reported as an important odorant in red wines of different grape varieties [42].

Other norisoprenoids identified in the wines were vitispirane (camphor or eucalyptus odour), α-ionone (violet-floral odour), 3-oxo-α-ionol (spicy odour), and its oxidation products the 3-hydroxy-7,8-dihydro-β-ionol. Most of them were found in higher concentrations in wines from sonicated grapes and macerated 48 h. However, this effect was less evident in the wines from sonicated grapes with 72 h of maceration, especially at 28 kHz, as it happened in the case of terpenes. Sonication at higher frequencies seems to cause degradation of these compounds [34].

Tartian et al. [9], obtained lower values of terpenes in wine from sonicated grapes at 35 kHz for 15 min compared to the values obtained by grape cryomaceration. Must thermovinification at high temperature also negatively affected the terpene content [11]. In Monastrell wines from grapes sonicated at 28 kHz, no increase in terpenes or nor-isoprenoids was observed [24,26]. However, wines from Shiraz grapes subjected to the same frequency, showed an increase of 30 % in their content of nerol and geraniol [32]. These results highlight the importance of the grape variety and US treatment conditions in the chemical changes produced in wine by sonication.

Terpenes and nor-isoprenoids can be found in the berry as non-volatile glycosides at the beginning of the winemaking process, but during skin maceration and fermentation they can be released by enzymatic or acid hydrolysis. The bound fraction of terpenes and nor-isoprenoids was slightly modified by sonication treatments as happened in the musts (Table 2). However, some individual compounds (geraniol, geranic acid, 3-oxo-α-ionol and 6,7-dihydro-7,8-dihydro-3-oxo-α-ionol) showed higher concentrations in the wines from sonicated grapes at 28 kHz and 72 h of skin maceration, which indicates a greater effect on the extraction of bound compounds under these conditions.

TDN was only identified in the bound fraction, probably because this compound tends to reach their highest concentrations after wine bottle or during the aging process, usually via acid hydrolysis [43].

Regarding free benzene compounds (Table 1), many of them had higher amounts in wines than in musts (4-vinylguaiacol, guaiacol, syringol), which shows an important cinnamyl-esterase activity of yeasts during fermentation.

Guaiacol (smoky, sweet, medicinal aroma) was found above its olfactory threshold in all wines (10 μg/L) [41], being more abundant in wines sonicated at 20 kHz with 72 h of skin maceration. 4-Vinylguaiacol (spicy aroma), also exceeded its perception threshold (40 μg/L) [41], but no significant differences were found between samples. Other benzenic compounds such as benzaldehyde, vanillin and their derivatives obtained lower values in wines sonicated at a higher frequency (28 kHz).

Bautista-Ortín et al. [24], reported lower amounts of benzyl alcohol in wines from grapes treated with ultrasound at pilot scale (28 kHz), while volatile phenols (4-ethylphenol and 4-ethylguaicol), which can negatively influence the aroma of these wines, increased.

The total glycosidically-bound benzene compounds (Table 2) were quantitatively lower in wines from sonicated grapes. Just some individual bound compounds (4-vinyl- guaiacol, vanillin and ethyl vanillate) increased in the wines made from sonicated grapes at 28 kHz and 72 h of maceration. This fact supports the hypothesis of the possible release of aglycones from their precursors due to sonication [33].

Regarding the effect of maceration time on the content of varietal compounds, the differences found in both the free and bound fraction were not statistically significant in most cases. This fact indicates that, although most of compounds are more abundant in the grape skin, possibly they are transferred to the must during the first days of maceration.

Broadly, it could be said that grape sonication at both frequencies and 48 h maceration was more effective when talking about varietal volatile extraction or generation, remarkably in the case of C_6_ alcohols, terpenes and nor-isoprenoids. Treatment at 28 kHz with 72 h of skin maceration adversely affected certain sensorially relevant compounds in free form.

It is difficult to establish a precise trend in the behavior of varietal compounds in wines due to the sonication treatment because the effects of sonication can be diverse. Extraction of free or bound compounds from the grape or the breaking of bound compounds can be favored by sonication. However, changes in the permeability of grape cell membranes due to US can selectively change their permeability so that not all compounds exhibit the same behavior [37].

The wines sonicated with 48 h of maceration showed a quantity of varietal compounds equal to or greater than the wines with a traditional maceration of 7 days, so from the point of view of the volatile composition, the maceration times could be considerably reduced.

### 2.3. Effect of Ultrasound Treatment on Volatile Compounds Formed during Alcoholic Fermentation of Control and Wines from Sonicated Grapes

Fermentation compounds are the main contributors to the aroma of young wines, especially those from non-aromatic grape varieties, and constitute the base of wine aroma [38].

**Figure 2:**
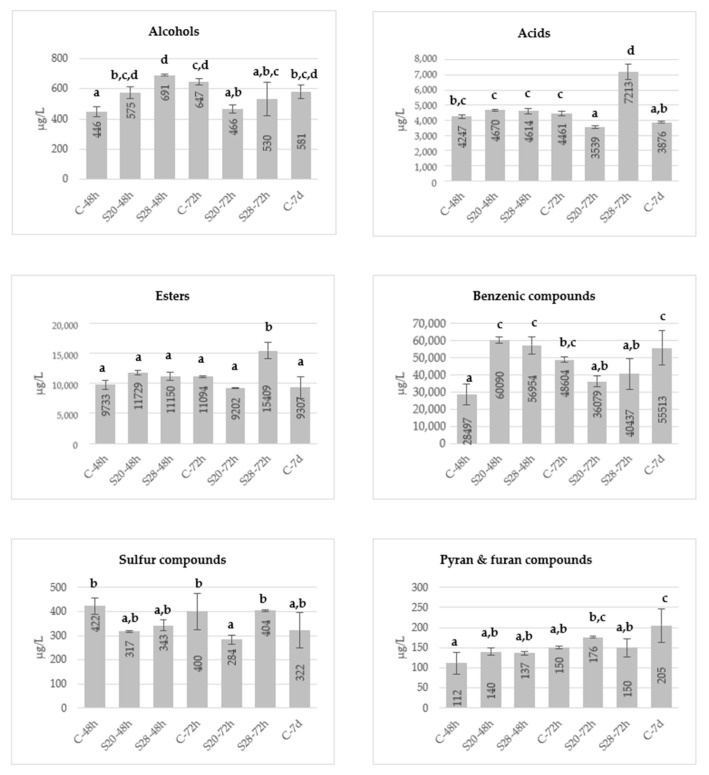
Mean concentrations (μg/L) of main volatile compounds formed during fermentation regarding control and wines made from sonicated grapes. ^a,b,c,d^ Different superscripts denoted significant differences according to the Student-Newman-Keuls test at *p* ˂ 0.05. C-48: control wine with 48 h of skin maceration; C-72: control wine with 72 h of skin maceration; S20-48: wine from grape sonicated at 20 kHz with 48 h of skin maceration; S28-72: wine from grape sonicated at 28 kHz with 72 h of skin maceration; C-7d: control wine with 7 days of skin maceration.

Figure 2 shows the total concentrations of the main groups of volatile compounds formed during alcoholic fermentation in control and treated wines. In comparison with control wines, the wines obtained from grapes treated by ultrasound had similar or higher concentrations in all the groups of volatile compounds. In the case of acids and esters, the increase in wines made from sonicated grapes at 28 kHz macerated 72 h was quite remarkable. These results could be related to a positive effect of sonication (18–30 kHz) in the fermentation process since it has been described a positive effect of US on the growth of *Saccharomyces cerevisiae*, with a reduction in fermentation time and a higher production of volatile compounds [19].

Fatty acids and esters come from yeast metabolism. They slightly contribute to their overall aroma due to their high olfactory thresholds, although they can be in high concentrations in wines. However, some esters have been described as important odorants in wines [42]. Short and medium chain fatty acid esters such as ethyl butanoate, ethyl hexanoate and ethyl octanoate can provide fruity aromas to the wines (strawberry, apple, fruity, sweet). These compounds were found at concentrations above their perception thresholds in all the wines studied, especially in wines obtained from sonicated grapes (Supplementary Materials Table S3), so their influence on the wine aroma will be of great relevance.

The same effect was observed in the case of ethyl esters of hydroxyacids, mainly in wines from sonicated grapes at 28 kHz and 72 h of skin maceration. The influence of ethyl 2-hydroxy-3-methylbutyrate and ethyl 2-hydroxy-4-methylpentanoate on the blackberry aroma of red wines has been demonstrated, even at concentrations below their perception thresholds [44,45].

However, the concentration of acetates (isoamyl acetate, hexyl acetate) decreased or remained constant in wines made from sonicated grapes at both maceration times. In studies carried out by other authors, when US is applied during the grape maceration, lower production of acetates was observed, while the total esters remained constant or increased, depending on the treatment conditions [26,32,46].

With regard to alcohols and total benzene compounds, the sonication effect was positive in wines treated at both frequencies macerated 48 h, but few changes were observed in wines from sonicated grapes macerated 72 h (Figure 2).

Although most aliphatic alcohols are not very relevant from the sensory point of view, unsaturated alcohols are characterized by presenting lower thresholds. Specifically, 1-octen-3-ol, presents a “mushroom like” aroma and was found in all samples above its perception threshold (1 μg/L) [47], so it could negatively influence the aroma of wines. The lowest concentrations were found in the sample sonicated at 28 kHz with 72 h of skin maceration (Table S3, Supplementary Materials).

Among the benzene compounds, the most abundant in all samples were 2-phenyl ethanol, benzeneacetaldehyde and 2-phenylethyl acetate. They present pleasant aromas (flowery, sweety, rose aromas) and were found in all samples above their perception threshold. All of them were positively affected by sonication at 48 h of skin maceration Martínez-Pérez et al. [26] observed similar amounts of total alcohols, but lower contents of 2-phenylethanol and benzyl alcohol in Monastrell wines from grape sonicated at 28 kHz.

Within the group of pyrans and furans, three lactones (γ-butyrolactone, γ-nonalactone, pantolactone) were quantified in tested wines. Lactones can contribute positively to the aroma of wines, and some of them have stood out as important odorants [38]. γ-Nonalactone, with a coconut-like aroma, has a low odor threshold (30 μg/L) [48]. No significant differences were detected due to maceration or ultrasound treatment (Figure 2).

The volatile sulfur compounds identified in the samples come from amino acids metabolism and could generate off-flavours. 2-Mercaptoethanol and 3-(methylthio)-1-propanol (methionol) are the most abundant sulfur compounds in young wines [49]. Methionol presented the highest concentrations in all the wines analyzed but his olfactory threshold was not exceeded in any of them. Sonication did not produce significant changes in the concentration of these compounds, although a small decrease was observed in some sonicated samples (Figure 2).

An effect of the maceration time was also observed in the control wines, a significant increase in alcohols, benzene compounds, furans and pyrans was reported after 72 h of maceration. On the other hand, the sonication of crushed grapes also increased the amount of most of the groups of volatile compounds generated in fermentation, obtaining amounts similar or higher than those found in wines with traditional 7-day fermentation.

As described above, ultrasound treatment of grapes results in greater extraction of compounds from cells. These compounds (amino acids, fatty acids…) can be used by yeasts as precursors for the formation of aroma compounds, justifying further formation in the wines made from sonicated grapes. It has also been observed that the sonication treatment accelerates the fermentation process making it more efficient, with the consequent increase in the amount of metabolites produced by yeasts [25,32].

### 2.4. Sensorial Analysis of Control and Wines from Sonicated Grapes

Modification of wine flavor and aroma profile due to sonication has been described by some authors [22]. To evaluate the effect of the ultrasound treatment on the sensory perception of Monastrell wines, the sensory analysis was carried out with a panel of expert tasters. The following olfactory attributes were selected as those that best defined the aroma of the wines: red berry, ripened fruit, spicy, floral, tobacco and herbaceous. Some taste attributes were also evaluated: body, astringency and aftertaste intensity, since they can be influenced by the US treatment of wines and therefore, they could be of interest to estimate the organoleptic quality of the wines [26].

**Figure 3:**
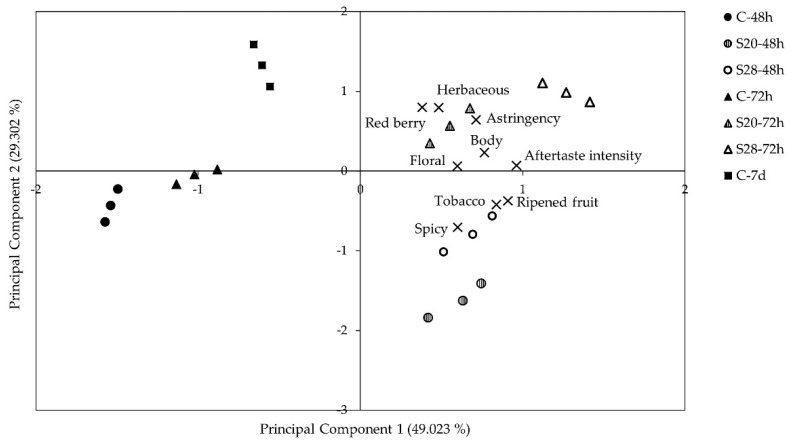
Distribution in the space of control and wines made from sonicated grapes, joint with their sensorial attributes. C-48: control wine with 48 h of skin maceration; C-72: control wine with 72 h of skin maceration; S20-48: wine from grape sonicated at 20 kHz with 48 h of skin maceration; S28-72: wine from grape sonicated at 28 kHz with 72 h of skin maceration; C-7d: control wine with 7 days of skin maceration.

Figure 3 presents the principal component biplot, illustrating the simultaneous projection of wines and their sensorial descriptors. The first two principal components explained 78.3% of the total variance. PC1 and PC2 accounted for 49.0% and 29.3% of the variance between samples, respectively. The first principal component clearly separated the control wines with 48 h, 72 h and 7 days of maceration from the wines treated with sonication, which were in the positive part of this axis, indicating a strong effect of sonication on the profile of the obtained wines.

The wines from grapes sonicated at 20 kHz and 28 kHz were characterized by having higher scores in all the evaluated attributes, compared with their control wines, and the application of the US at the higher frequency slightly increasing all the scores. Those made with sonicated grapes and with 72 h of maceration obtained higher scores in the attributes: berry fruit, floral, and herbaceous aroma, also presenting greater body, astringency, and aftertaste intensity. These attributes positively correlated with PC2 were also important in wines with traditional production (7 days of maceration with skins).

Martinez-Pérez et al. [26], observed that Monastrell wines from grapes treated with US on pilot scale, showed higher astringency, quality and aromatic intensity than control wines. The greater astringency and body might be due to the increase in tannin extraction previously described in wines from sonicated grapes of this variety [24].

Berry fruit aroma has been associated to the content of some fatty acid ethyl esters (ethyl butanoate, ethyl hexanoate), as well as hydroxy acid esters [44,45]. The higher content of these compounds in wines from sonicated grapes (Table S3, Supplementary Materials) could justify their higher scores in this attribute. On the other hand, many volatile compounds have sensory descriptors related to the “floral” attribute (linalool, a-ionone, 2-phenylethanol), of which 2-phenylethanol was found in the highest concentration in the samples, especially in US treated.

Additionally, US treated wines with 48 h of maceration stood out for their aroma of tobacco, ripened fruit and spicy. These wines also had higher concentrations of 3-oxo-α-ionol, a compound that has been described as possible contributors to tobacco-like aroma [50], as well as benzene compounds such as guaiacol, 4-vinylguaiacol and vanillin related to the spicy and vanilla aromas [51] (Table 1).

The effect of sonication on the sensory perception of wines differs according to the studies carried out, depending on the conditions and type of US treatment. Ruiz-Rodriguez et al. [32], observed better scores in fruity and floral aroma in wines from Syrah grapes sonicated during maceration, while Cui et al. [31], reported more complex aromas only in white wines sonicated for short times. The sensory evaluation of red wines elaborated with thermovinification and with US treatment was similar, obtaining greater aromatic complexity and perception of fruit aromas than the control wine [46].

Gracin et al. [52] observed that ultrasound treatment at 24 kHz through a continuous flow reactor caused negative changes in wine sensorial properties, with the formation of oxidized aromas and negative burnt and smoky smells. However, in our case, the tasters did not detect any unpleasant flavour or uncharacteristic attribute of the red wines of this grape variety.

In any case, when selecting the best maceration or US treatment conditions in red wine, the color parameters should also be considered. A previous study carried out by the same authors established that wines from sonicated grapes and 72 h of skin maceration were chromatically very similar to control wines with 7 days of skin contact, which would significantly reduce maceration times [25].

## 3. Materials and Methods

### 3.1. Grape Samples

Monastrell red grapes were harvested from a commercial vineyard in Jumilla (Murcia, Spain) when they reached 14° Baumé, being quickly transported to the winery for their processing.

### 3.2. Winemaking Process

Grapes (1400 kg) were destemmed, crushed and divided in three batches. One batch was used for control vinification (C). The other two batches were immediately treated with a pilot-scale power ultrasound system (MiniPerseo, Agrovin S.A., Alcázar de San Juan, Spain) using two different frequencies, 20 kHz (S20) and 28 kHz (S28). This system is capable of treating up to 400 kg of crushed grapes per hour and operated at 2500 W with a power density of 8 W/cm^2^. Alcoholic fermentation-macerations were carried out in 50 kg stainless steel tanks, which were filled with the control or ultrasound-treated crushed grapes, maintaining the same solid/liquid ratio in each tank. Total acidity was corrected to 5.5 g/L (tartaric acid) and 20 g of selected dry yeast per 100 kg of crushed grapes were added (Viniferm CT007, Agrovin). Two different skin maceration times were tested for the sonicated grapes at each ultrasound frequency used: 48 h (S20-48 and S28-48) and 72 h (S20-72 and S28-72). While for the control wines were tested three times, 48 h and 72 h (C-48h and C-72h), and 7 days (C-7d). Each vinification was executed in triplicate. The tanks were maintained at 23 ± 2 ℃. Two punching downs were carried out every day during maceration period. At the end of this period, the wines were pressed in a 75 L pneumatic press. Free-run and press wines were combined and stored at room temperature until the end of alcoholic fermentation. When the fermentation process finished, wines were cold-stabilised at 2 °C for one month, racked from lees and bottled. Wines were analyzed after bottling.

### 3.3. Isolation of Free and Glycosidically-Bound Minor Volatile Compounds

Before analysis execution, wines were centrifuged at 4 °C (10,000 rpm, 10 min) (Avanti Centrifuge J26-XP, Beckman Coulter, Indianapolis, IN, USA), then a filtration step was carried out through a 1.2 μm glass fiber membrane (Fisherbrand, Thermo Fisher Scientific, Inc., Waltham, MA, USA). Minor volatile compounds (both free and glycosidically-bound) of the Monastrell wines were extracted in duplicate by solid phase extraction (SPE) following the method used by Sanchez-Palomo et al. [3] with minor modifications.

#### 3.3.1. Solid Phase Extraction of Free Volatile Compounds

The SPE step was performed using 500 mg styrene-divinylbenzene cartridges, (Lichrolut EN, Merck KGaA, Darmstadt, Germany). The cartridges were previously conditioned by flowing (2 mL/min) 10 mL of dichloromethane, followed by 5 mL of methanol, and 10 mL of 10% (*v/v*) aqueous ethanol. Then, 100 mL of wine together with 40 μL of 4-nonanol added as internal standard (1 g/L) were passed through the cartridge. Non-volatile hydrophilic compounds were washed out of the cartridges using 50 mL of bidistilled Milli Q Plus water and free minor volatile compounds were eluted with 10 mL of dichloromethane. The extracts were concentrated to an approximate volume of 200 μL under a gentle stream of nitrogen and carefully stored in a freezer (−20 °C) until their chromatographic analysis.

#### 3.3.2. Solid Phase Extraction of Glycosidically-Bound Volatile Compounds

Bound compounds were eluted from the same cartridge used in the previous step by using 25 mL of ethyl acetate: methanol (90:10). Extracts were evaporated at 55 °C to almost dryness (≈1 mL) under vacuum (290 mBar) and complete dryness was achieved by a gentle stream of nitrogen gas. Dry extracts, widely distributed in the flask walls, were accurately re-dissolved with 1 mL of methanol, and evaporated again under a gentle stream of nitrogen gas. The enzymatic hydrolysis was performed by adding 2 mL of phosphate-citrate buffer (0.1–0.2 M; pH = 5) containing 0.20 μL/mL of a commercial enzyme “Trenolin Bouquet PLUS” (ERBSLÖH, ERBSLÖH Geisenheim AG, Geisenheim, Germany). The extracts were incubated at 40 °C for 16 h. Then, 25 mL of synthetic wine (12% ethanol, pH = 3.5) were added to the flask containing the released aglycones, and a new SPE step was carried out to extract the released volatile compounds using 200 mg styrene-divinylbenzene cartridges, (Lichrolut EN Merck KGaA). The cartridges were previously conditioned by flowing (2 mL/min) 5 mL of dichloromethane, followed by 2.5 mL of methanol, and 5 mL of 10% (*v/v*) aqueous ethanol. Then, 27 mL of sample was passed through the cartridge. Non-volatile hydrophilic compounds were washed out of the cartridges using 25 mL of bidistilled Milli Q Plus water, and minor-released volatile compounds were eluted with 5 mL of dichloromethane. The extracts were concentrated to an approximate volume of 200 μL under a gentle stream of nitrogen and carefully stored in a freezer (−20 °C) until their chromatographic analysis.

### 3.4. Gas Chromatography-Mass Spectrometry (GC-MS)

One microliter (1 µL) of each extract was injected in splitless mode (0.30 min for free volatile compounds and 0.60 min for glycosidically-bound volatile compounds) into an Agilent 6890 GC System equipped with an Agilent 5973 Inert Mass Selective Detector and a polar DB-WAX ultra-inert column (60 m × 0.25 mm × 0.25 µm) (Agilent Technologies, Waldbronn, Germany). The carrier gas was helium at 1 mL/min. The column temperature was programmed as follows: 70 °C for 5 min, raised at 1 °C/min to 90 °C (10 min) and then increased 2 °C/min to 210 °C (40 min). The injector temperature was 250 °C. The MS worked in the electron impact mode with an electron energy of 70 eV, the ion source temperature was 230 °C and the scanning was made from 45 to 550 a.m.u.

Identification of the volatile compounds was executed by comparison with trustworthy standards from Sigma-Aldrich (Tres Cantos, Madrid, Spain). Tentative identification of compounds for which it was not possible to find volatile references was carried out by comparison of their mass spectra with spectral data from the Wiley G 1035 A, NBS75K and NIST14 libraries along with linear retention index (LRI) comparison. The response factor of each compound was calculated by injecting commercial standards as the relationship between the peak area and its concentration, both referred to the internal standard. Response factors of compounds presenting similar chemical structures were used for those whose commercial standards were not available, (hydroxylinalool was quantified as linalool; hydroxycitronellol as citronellol; geranyl acetate as trans-geraniol; 3-oxo-α-ionol, 3-hydroxy-7,8-dihydro β-ionol, 2,3-dehydro-4-oxo-β-ionol as α-ionol; TDN and vitispirane as β-damascenone; benzenealcetaldehyde as acetaldehyde; 4-methyl-1-pentanol as 3-methyl-1-pentanol; 3-methylbutanoic acid and 2-methylpropanoic acid as butanoic acid; 2-hexenoic acid as hexanoic acid; ethyl 2-hydroxy-3-methylbutyrate, methyl 2-hydroxy-4-methylpentanoate, and ethyl 4-hydroxybutyrate were quantified as ethyl 3-hydroxybutyrate; monomethyl succinate as ethyl succinate; ethyl 3-(methylthio)-propionate and 3-(methylthio)-propanoic acid as 3-(methylthio)-propanol). Results were expressed in micrograms per liter (µg/L).

### 3.5. Sensory Descriptive Analysis

Monastrell red wines were tasted by a group of expert laboratory staff experienced in sensory analysis. The assessment took place in a standard sensory analysis chamber (ISO 8589:2007) equipped with separate booths and wine-tasting glasses (ISO 3591:1997) covered with a watch-glass to minimize the escape of volatile compounds. Wines were sniffed and tasted by eight judges, which generated sensory terms individually, agreeing on the following descriptors: red berry, ripened fruit, spicy, floral, tobacco, herbaceous, body, astringency, and aftertaste intensity. The panelists used a 10 cm unstructured scale to rate the intensity of each attribute. The left extreme of the scale indicated a null intensity of the descriptor and the right extreme the maximum value.

### 3.6. Statistical Analysis

The statistical analysis was executed using the IBM SPSS statistics v.24.0 for Windows statistical package (IBM, Madrid, Spain). The volatile data set was analyzed with the Student-Newman-Keuls test, a multiple-step comparison procedure used to identify samples that showed significant differences due to maceration time or ultrasound treatment. Principal component analysis (PCA) was also carried out to simplify the complexity of the data sets and spotlight the main contributors to the variance amongst the different samples studied.

## 4. Conclusions

These results indicate that sonication affects the extraction of varietal compounds, causing an increase in the extraction of compounds as well as the breakdown of the grape precursors. In this way, the application of US to crushed grapes led to an increase in most of varietal compounds in free form in the musts, while those that remained as glycosidically bound fraction were less affected by this treatment. In the resulting wines there was a positive effect of the US treatment on C6 alcohols, terpenes and norisoprenoids (all varietal compounds) at both frequencies, especially with the shorter maceration time.

Fermentation compounds were also affected by grape sonication, showing a significant increase in acids and esters at the frequency of 28 kHz as used and skin maceration lasted 72 h. These differences are unmistakable in the sensory analysis of wines, those wines made from sonicated grapes and with 72 h of skin maceration presented the highest scores in berry fruit, floral and herbaceous aromas, also presenting higher astringency, body and aftertaste intensity, being the most similar to control wines with 7 days of maceration. Therefore, these sonication conditions, as previously observed for their chromatic characteristics [25], can lead to wines that resemble the characteristics of traditionally produced wines, even with improved sensory scores, but with reduced maceration times.

## Figures and Tables

**Table 1:** Mean concentrations (μg/L) and relative standard deviations (*n* = 3) of volatile compounds (free fraction) in control and wines made with sonicated grapes with different maceration periods.

Volatile Compounds	C-48	S20-48	S28-48	C-72	S20-72	S28-72	C-7d
	Mean ± SD	Mean ± SD	Mean ± SD	Mean ± SD	Mean ± SD	Mean ± SD	Mean ± SD
1-hexanol	1678 ± 239 ^a^	2159 ± 9 ^b,c^	2395 ± 57 ^c^	2001 ± 133 ^a,b,c^	1819 ± 88 ^a,b^	1784 ± 159 ^a,b^	2003 ± 298 ^a,b,c^
*cis*-3-hexen-1-ol	65.3 ± 9.1 ^a^	84.8 ± 0.4 ^a^	80.7 ± 7.0 ^a^	75.8 ± 5.8 ^a^	68.1 ± 2.8 ^a^	105 ± 5 ^b^	74.1 ± 15.7 ^a^
*trans*-3-hexen-1-ol	24.3 ± 1.2 ^a^	34.0 ± 1.4 ^b,c^	29.3 ± 3.7 ^a,b^	21.2 ± 1.0 ^a^	22.6 ± 2.1 ^a^	40.8 ± 8.5 ^c^	22.7 ± 4.6 ^a^
*cis*-2-hexen-1ol	8.5 ± 0.5 ^a^	10.5 ± 0.8 ^b^	8.4 ± 0.6 ^a^	10.1 ± 0.3 ^b^	8.0 ± 0.2 ^a^	10.2 ± 0.4 ^b^	13.7 ± 0.6 ^c^
*trans*-2-hexen-1ol	13.0 ± 1.5 ^a^	21.3 ± 0.5 ^b,c^	23.6 ± 2.7 ^c^	18.6 ± 3.4 ^b^	16.1 ± 0.6 ^a,b^	17.3 ± 2.3 ^a,b^	18.4 ± 2.7 ^b^
**Σ C6-alcohols**	**1789 ± 248 ^a^**	**2310 ± 10 ^b,c^**	**2537 ± 71 ^c^**	**2127 ± 138 ^a,b,c^**	**1934 ± 84 ^a,b^**	**1958 ± 164 ^a,b^**	**2132 ± 322 ^a,b,c^**
linalool	12.4 ± 1.3 ^b^	13.4 ± 0.6 ^b,c^	14.8 ± 0.2 ^c,d^	15.5 ± 1.0 ^d^	13.0 ± 0.0 ^b,c^	3.9 ± 0.5 ^a^	14.4 ± 1.1 ^c,d^
4-terpineol	13.8 ± 3.2 ^b^	14.2 ± 0.8 ^b^	13.8 ± 0.8 ^b^	20.9 ± 1.8 ^c^	13.4 ± 1.2 ^b^	5.3 ± 0.3 ^a^	15.3 ± 0.3 ^b^
α-terpineol	6.4 ± 1.1 ^a^	6.3 ± 0.6 ^a^	6.3 ± 0.7 ^a^	6.3 ± 0.6 ^a^	8.6 ± 3.2 ^a^	6.1 ± 1.3 ^a^	8.0 ± 1.2 ^a^
citronellol	20.9 ± 5.5 ^b,c^	23.7 ± 4.4 ^b,c^	21.8 ± 1.2 ^b,c^	24.3 ± 1.0 ^b,c^	29.5 ± 6.7 ^c^	7.8 ± 0.3 ^a^	17.5 ± 0.0 ^b^
*trans*-geraniol	4.9 ± 0.5 ^a^	5.1 ± 0.2 ^a^	4.3 ± 0.3 ^a^	6.8 ± 1.5 ^b^	4.3 ± 0.4 ^a^	7.8 ± 0.2 ^b^	6.5 ± 0.5 ^b^
hydroxylinalool	7.4 ± 1.2 ^c^	4.7 ± 0.5 ^b^	5.9 ± 0.4 ^b,c^	6.4 ± 0.3 ^b,c^	4.7 ± 0.6 ^b^	2.8 ± 0.2 ^a^	5.9 ± 1.0 ^b,c^
hydroxycitronellol	4.0 ± 0.4 ^b,c^	3.6 ± 0.1 ^a,b,c^	4.3 ± 0.2 ^c^	4.5 ± 0.5 ^c^	2.9 ± 0.6 ^a,b^	2.7 ± 0.2 ^a^	4.5 ± 1.1 ^c^
geranyl acetate	19.3 ± 2.7 ^d^	15.2 ± 0.8 ^c^	12.7 ± 3.1 ^b,c^	2.2 ± 0.1 ^a^	15.7 ± 0.2 ^c^	10.2 ± 0.0 ^b^	13.1 ± 1.5 ^b,c^
α-ionone	5.4 ± 0.3 ^d^	1.0 ± 0.3 ^a^	1.1 ± 0.0 ^a^	1.4 ± 0.1 ^a,b^	2.4 ± 0.3 ^c^	1.0 ± 0.0 ^a^	1.6 ± 0.1 ^b^
vitispirane	3.0 ± 1.2 ^a^	11.1 ± 0.3 ^c^	5.2 ± 1.0 ^b^	2.3 ± 0.5 ^a^	2.4 ± 1.1 ^a^	ND	0.60 ± 1.06 ^c^
β-damascenone	7.3 ± 0.2 ^b^	7.1 ± 0.2 ^b^	7.3 ± 0.1 ^b^	7.3 ± 1.4 ^b^	5.6 ± 0.0 ^b^	2.6 ± 1.3 ^c^	6.2 ± 0.0 ^b^
3-oxo-α-ionol	7.3 ± 0.4 ^a^	20.0 ± 11.5 ^b,c^	13.0 ± 0.5 ^a,b^	10.4 ± 0.9 ^a,b^	8.3 ± 1.0 ^a,b^	25.1 ± 3.2 ^c^	15.2 ± 3.2 ^a,b,c^
3-hydroxy-7,8-dihydro β-ionol	46.6 ± 7.3 ^b^	69.4 ± 4.0 ^c^	61.0 ± 0.6 ^c^	29.5 ± 6.8 ^a^	29.1 ± 4.6 ^a^	27.7 ± 0.6 ^a^	59.7 ± 10.4 ^c^
2,3-dehydro-4-oxo-β-ionol	1.4 ± 0.4 ^a^	ND	ND	1.5 ± 0.2 ^a^	1.5 ± 0.0 ^a^	1.9 ± 0.2 ^b^	ND
**Σ Terpenes and norisoprenoids**	**160 ± 1 ^b,c^**	**195 ± 12 ^d^**	**171 ± 8 ^c^**	**139 ± 8 ^b^**	**141 ± 7 ^b^**	**104 ± 5 ^a^**	**171 ± 19 ^c^**
benzaldehyde	57.2 ± 4.9 ^b,c^	66.6 ± 0.0 ^c^	14.2 ± 3.1 ^a^	38.3 ± 25.1 ^b^	43.0 ± 6.4b	8.0 ± 1.3 ^a^	14.4 ± 0.4 ^a^
guaiacol	236 ± 75 ^b^	220 ± 1 ^b^	175 ± 11 ^a,b^	101 ± 10 ^a^	462 ± 51 ^c^	134 ± 10 ^a^	180 ± 30 ^a,b^
benzyl alcohol	208 ± 10 ^a^	322 ± 0 ^b^	298 ± 38 ^b^	312 ± 43 ^b^	275 ± 2 ^b^	261 ± 27 ^b^	456 ± 14 ^c^
4-vinylguaiacol	248 ± 62 ^a^	385 ± 2 ^a^	226 ± 168 ^a^	216 ± 44 ^a^	204 ± 18 ^a^	245 ± 31 ^a^	388 ± 58 ^a^
syringol	613 ± 122 ^a^	459 ± 25 ^a^	415 ± 16 ^a^	171 ± 21 ^a^	607 ± 326 ^a^	745 ± 203 ^a^	464 ± 94 ^a^
vanillin	16.8 ± 4.8 ^b^	17.8 ± 2.7 ^b,c^	11.8 ± 3.2 ^a,b^	23.7 ± 2.4 ^d^	18.3 ± 0.8 ^b,c^	7.1 ± 2.8 ^a^	7.3 ± 1.3 ^a^
methyl vanillate	11.5 ± 1.1 ^a^	13.0 ± 0.3 ^a^	12.6 ± 0.0 ^a^	11.7 ± 1.5 ^a^	10.0 ± 0.5 ^a^	10.8 ± 0.1 ^a^	13.0 ± 2.8 ^a^
ethyl vanillate	154 ± 21 ^b,c^	164 ± 11 ^c^	131 ± 10 ^b,c^	137 ± 25 ^b,c^	119 ± 1.8 ^b^	52.2 ± 5.3 ^a^	162 ± 15 ^c^
**Σ Benzenic compounds**	**1545 ± 263** **^a^**	**1649 ± 35 ^a^**	**1285 ± 229 ^a^**	**1238 ± 66** **^a^**	**1738 ± 406 ^a^**	**1464 ± 156 ^a^**	**1715 ±3 7 ^a^**

^a,b,c,d^ Different superscripts in the same row denoted significant differences according to the Student-Newman-Keuls test at *p* ˂ 0.05. C-48: control wine with 48 h of skin maceration; C-72: control wine with 72 h of skin maceration; S20-48: wine from grape sonicated at 20 kHz with 48 h of skin maceration; S28-72: wine from grape sonicated at 28 kHz with 72 h of skin maceration; C-7d: control wine with 7 days of skin maceration.

**Table 2:** Mean concentrations (μg/L) and relative standard deviations (*n* = 3) of volatile compounds (bound fraction) in control and wines made with sonicated grapes with different maceration periods.

Volatile Compounds	C-48	S20-48	S28-48	C-72	S20-72	S28-72	C-7d
	Mean ± SD	Mean ± SD	Mean ± SD	Mean ± SD	Mean ± SD	Mean ± SD	Mean ± SD
2-hexenal	1.5 ± 0.2 ^b^	0.33 ± 0.05 ^a^	ND	1.0 ± 0.1 ^b^	2.3 ± 0.6 ^c^	1.4 ± 0.2 ^b^	ND
1-hexanol	13.1 ± 1.0 ^c^	16.7 ± 0.8 ^a^	4.9 ± 0.6 ^a^	11.2 ± 2.5 ^b,c^	9.6 ± 1.4 ^b^	9.5 ± 1.7 ^b^	5.2 ± 1.4 ^a^
*cis*-2-hexen-1-ol	2.6 ± 0.2 ^c^	0.94 ± 0.03 ^a^	0.83 ± 0.04 ^a^	2.2 ± 0.5 ^b,c^	1.8 ± 0.3 ^b^	1.6 ± 0.3 ^b^	1.1 ± 0.3 ^a^
**Σ C6-alcohols and aldehydes**	**17.2 ± 1.3 ^c,d^**	**18.0 ± 1.0 ^d^**	**5.8 ± 0.6 ^a^**	**14.4 ± 2.9 ^b,c,d^**	**16.6 ± 2.1 ^b,c^**	**12.8 ± 1.1 ^b^**	**6.2 ± 1.7 ^a^**
nerol	1.4 ± 0.1 ^b^	0.61 ± 0.00 ^a^	0.49 ± 0.09 ^a^	1.6 ± 0.2 ^b^	1.6 ± 0.3 ^b^	1.4 ± 0.1 ^b^	0.62 ± 0.10 ^a^
geraniol	3.4 ± 0.2 ^a,b^	1.9 ± 0.4 ^a^	1.8 ± 0.1 ^a^	5.1 ± 1.6 ^b^	4.2 ± 0.8 ^b^	4.2 ± 1.1 ^b^	1.5 ± 0.1 ^a^
hydroxylinalool	0.98 ± 0.15 ^a^	0.67 ± 0.06 ^a^	0.55 ± 0.08 ^a^	1.1 ± 0.3 ^a^	0.95 ± 0.08 ^a^	2.1 ± 1.2 ^b^	0.72 ± 0.18 ^a^
geranic acid	3.6 ± 0.4 ^a^	3.0 ± 0.4 ^a^	5.2 ± 1.9 ^a^	4.1 ± 0.4 ^a^	5.2 ± 2.1 ^a^	11.1 ± 1.5 ^b^	3.3 ± 0.1 ^a^
geranyl acetate	4.4 ± 0.7 ^a^	3.7 ± 0.3 ^a^	5.1 ± 1.2 ^a^	4.3 ± 0.3 ^a^	3.8 ± 0.1 ^a^	7.1 ± 0.7 ^a^	7.2 ± 1.7 ^a^
TDN	ND	0.30 ± 0.01 ^b^	0.37 ± 0.06 ^b^	0.08 ± 0.01 ^a^	0.03 ± 0.00 ^a^	0.05 ± 0.05 ^a^	0.35 ± 0.04 ^b^
α-ionone	1.2 ± 0.0 ^a^	1.1 ± 0.3 ^a^	1.2 ± 0.1 ^a^	ND	1.9 ± 0.3 ^a^	1.4 ± 0.4 ^a^	3.7 ± 1.1 ^b^
β-damascenone	0.93 ± 0.05 ^a,b^	0.84 ± 0.23 ^a,b^	0.60 ± 0.06 ^a^	1.07 ± 0.16 ^b^	0.87 ± 0.24 ^a,b^	0.77 ± 0.01 ^a,b^	ND
3-oxo-α-ionol	1.8 ± 0.3 ^c^	1.0 ± 0.2 ^a,b^	0.63 ± 0.05 ^a^	1.7 ± 0.4 ^c^	1.4 ± 0.2 ^b,c^	4.8 ± 0.3 ^d^	0.63 ± 0.18 ^a^
6,7-dehydro-7,8-dihydro-3-oxo-α-ionol	9.0 ± 1.0 ^c,d^	2.6 ± 0.2 ^a^	4.7 ± 0.8 ^a,b,c^	9.8 ± 2.2 ^d^	7.7 ± 2.3 ^b,c,d^	22.4 ± 4.3^e^	3.8 ± 0.4 ^a,b^
**Σ Terpenes and norisoprenoids**	**26.7 ± 1.7 ^a^**	**15.7 ± 0.1 ^a^**	**20.6 ± 3.8 ^a^**	**28.9 ± 1.2 ^a^**	**27.5 ± 4.4 ^a^**	**55.2 ± 14.0 ^b^**	**21.7 ± 0.3 ^a^**
benzaldehyde	1.5 ± 0.2 ^a^	2.1 ± 0.6 ^b^	2.8 ± 0.2 ^c^	1.2 ± 0.2 ^a^	1.6 ± 0.1 ^a^	1.3 ± 0.2 ^a^	1.1 ± 0.2 ^a^
guaiacol	54.0 ± 4.4 ^c^	10.1 ± 2.4 ^a^	25.0 ± 4.0 ^b^	61.6 ± 11.4 ^c^	78.6 ± 4.7 ^d^	50.4 ± 0.8 ^c^	7.6 ± 0.8 ^a^
benzyl alcohol	8.1 ± 0.7 ^b,c^	5.4 ± 0.1 ^a^	4.8 ± 0.7 ^a^	9.2 ± 1.3 ^c^	7.9 ± 0.2 ^b,c^	7.6 ± 0.5 ^b^	7.3 ± 0.3 ^b^
2-phenylethanol	119 ± 7 ^b^	65.4 ± 15.1 ^b,c^	62.8 ± 8.2 ^b,c^	244 ± 44 ^d^	83.2 ± 14.9 ^b,c^	116 ± 31 ^b^	58.7 ± 6.2 ^a^
4-vinylguaiacol	55.8 ± 15.5 ^b^	14.0 ± 0.8 ^a^	18.6 ± 6.2 ^a^	53.0 ± 5.1 ^b^	51.3 ± 1.5 ^b^	98.7 ± 27.9 ^c^	11.4 ± 0.3 ^a^
syringol	57.7 ± 9.3 ^a,b^	44.9 ± 1.4 ^a^	58.4 ± 32.0 ^a,b^	168 ± 26 ^c^	73.5 ± 20.7 ^a,b^	138 ± 83 ^b,c^	29.3 ± 4.6 ^a^
vanillin	19.7 ± 0.6 ^a^	18.7 ± 1.4 ^a^	10.8 ± 0.9 ^a^	20.6 ± 3.7 ^a^	16.6 ± 2.3 ^a^	47.5 ± 4.2 ^b^	18.4 ± 8.4 ^a^
methyl vanillate	13.1 ± 2.1 ^a^	9.7 ± 1.0 ^a^	10.1 ± 0.4 ^a^	13.1 ± 3.9 ^a^	15.8 ± 0.8 ^a^	33.2 ± 10.6 ^b^	8.5 ± 2.6 ^a^
**Σ Benzenic compounds**	**329 ± 12 ^b^**	**170 ± 9 ^a^**	**193 ± 34 ^a^**	**572 ± 63 ^d^**	**329 ± 26 ^b^**	**493 ± 68 ^c^**	**142 ± 23 ^a^**

^a,b,c,d^ Different superscripts in the same row denoted significant differences according to the Student-Newman-Keuls test at *p* ˂ 0.05. C-48: control wine with 48 h of skin maceration; C-72: control wine with 72 h of skin maceration; S20-48: wine from grape sonicated at 20 kHz with 48 h of skin maceration; S28-72: wine from grape sonicated at 28 kHz with 72 h of skin maceration; C-7d: control wine with 7 days of skin maceration.

## Data Availability

Not available.

## Supplementary Materials

The following are available online. Table S1: Mean concentrations (μg/L) and relative standard deviations (*n* = 3) of volatile compounds (free fraction) in control and sonicated musts, Table S2: Mean concentrations (μg/L) and relative standard deviations (*n* = 3) of volatile compounds (bound fraction) in control and sonicated musts. Table S3: Mean concentrations (μg/L) and relative standard deviations (*n* = 3) of volatile compounds formed during the alcoholic fermentation in control and wines made from sonicated grapes with different maceration periods.
