# Supplementary material for: Effect of Power Ultrasound Treatment on Free and Glycosidically-Bound Volatile Compounds and the Sensorial Profile of Red Wines
Source: Molecules. 2021 Feb 23;26(4):1193. doi: 10.3390/molecules26041193 (PMC7926635; doi:10.3390/molecules26041193)
Supplement: Supplementary file 1 [file molecules-26-01193-s001.pdf]

Supplementary Table S1. Mean concentrations (µg/L) and relative standard deviations (n=3) of volatile compounds (free fraction) in control and sonicated musts.

Volatile compounds	C			S20			S28		
	**Mean**	**±**	**SD**	**Mean**	**±**	**SD**	**Mean**	**±**	**SD**
1-Hexanol	617	±	44[a]	764	±	33[b]	862	±	45[b]
cis-3-Hexen-1-ol	19.7	±	1.9[a]	29.0	±	2.1	32.4	±	0.8[b]
trans-3-Hexen-1-ol	5.8	±	1.4[a]	15.2	±	0.9	15.4	±	0.7[b]
cis-2-Hexen-1-ol	239	±	23	223	±	16	229	±	6
trans--Hexen-1-ol	11.3	±	0.4[b]	14.1	±	1.7[b]	15.2	±	0.9[a]
Benzaldehyde	19.5	±	0.7[a]	44.8	±	4.1[b]	49.9	±	1.2[b]
Linalool	2.6	±	0.7	1.8	±	0.3	3.1	±	0.0
1,4-Terpineol	12.9	±	1.0	10.8	±	0.2	11.6	±	1.8
trans-Geraniol	5.7	±	0.8[a]	9.0	±	1.3[ab]	11.4	±	1.4[b]
Guaiacol	3.5	±	0.3[a]	8.8	±	0.0[b]	8.0	±	0.2[c]
Benzylalcohol	50.0	±	1.0[a]	241	±	12[b]	278	±	8[c]
2-Phenylethanol	82.6	±	19.4[a]	140	±	13[b]	196	±	11[c]
4-Vinylguaiacol	12.9	±	1.3	14.6	±	2.6	15.6	±	5.0
3,7, dimethyl 1,7, octanediol	7.1	±	1.1	6.9	±	0.8	9.9	±	1.1
Vainillin	14.4	±	0.2[a]	22.2	±	0.6[b]	22.1	±	2.0

Values with different superscripts in the same row denoted significant differences according to the Student-Newman-Keuls test at $p < 0.05$. C: control must; S20: must from grape sonicated at 20 kHz; S28: must from grape sonicated at 28 kHz .

Supplementary Table 2. Mean concentrations (µg/L) and relative standard deviations (n=3) of volatile compounds (bound fraction) in control and sonicated musts.

Volatile compounds	C			S20			S28		
	**Mean**	**±**	**SD**	**Mean**	**±**	**SD**	**Mean**	**±**	**SD**
1-Hexanol	17.3	±	6.1	17.4	±	1.6	10.9	±	1.4
cis-3-Hexen-1-ol	1.1	±	0.1	1.2	±	0.1	0.87	±	0.31
trans-3-Hexen-1-ol	2.3	±	0.6	2.2	±	0.4	1.9	±	0.4
cis-2-Hexen-1-ol	9.2	±	2.8	13.3	±	1.2	8.5	±	0.6
trans--Hexen-1-ol	1.1	±	0.3	1.1	±	0.5	1.4	±	0.2
Benzaldehyde	4.7	±	0.5	4.8	±	0.4	4.5	±	1.8
Linalool	1.1	±	0.1[b]	0.63	±	0.06[a]	1.6	±	0.1[c]
1,4-Terpineol	2.0	±	0.2	2.6	±	0.6	2.5	±	0.5
trans-Geraniol	4.3	±	0.3	4.5	±	0.2	4.9	±	1.0
Guaiacol	1.8	±	0.3[a]	4.6	±	0.6[b]	5.6	±	0.4[c]
Benzylalcohol	45.6	±	5.6	41.4	±	3.0	38.5	±	6.6
2-Phenylethanol	44.5	±	15.7	29.6	±	0.3	30.5	±	7.5
4-Vinylguaiacol	41.5	±	3.5	54.2	±	16.5	41.4	±	11.6
1,7, octanediol, 3,7, dimethyl	2.2	±	0.3	1.8	±	0.6	2.9	±	0.9
Vainillin	19.1	±	1.3	24.9	±	1.4	27.2	±	8.5

Values with different superscripts in the same row denoted significant differences according to the Student-Newman-Keuls test at $p < 0.05$. C: control must; S20: must from grape sonicated at 20 kHz; S28: must from grape sonicated at 28 kHz.

Supplementary Table 3. Mean concentrations (μg/L) and relative standard deviations (n=3) of volatile compounds formed during the alcoholic fermentation in control and wines made from sonicated grapres with different maceration periods.

**Volatile compounds**	**C-48**	**S20-48**	**S28-48**	**C-72**	**S20-72**	**S28-72**	**C-7d**
	**Mean±SD**	**Mean±SD**	**Mean±SD**	**Mean±SD**	**Mean±SD**	**Mean±SD**	**Mean±SD**
butanol	36.1±7.3^{a}	56.0±1.9^{a,b}	69.9±18.6^{b,c}	89.5±6.0^{c}	49.7±9.6^{a,b}	84.0±16.2^{c}	81.5±0.8^{c}
3-methyl-1-butanol	56.6±2.8^{a,b}	88.5±1.9^{c,d}	73.3±12.1^{b,c}	87.4±6.3^{c,d}	62.9±4.8^{a,b}	51.2±4.4^{a}	93.1±14.0^{d}
4-mehtyl-1-pentanol	108±19^{b}	139±7^{b,c}	142±3^{b,c}	150±13^{c}	107±2.8^{b}	71.5±18.4^{a}	116±23^{b}
3-mehtyl-1-pentanol	178±14^{a}	211±24^{c,b}	200±7^{a,b}	228±4^{a,b}	165±11^{a}	259±69^{b}	165±14^{a}
3-octanol	2.9±0.2^{a,b}	3.1±0.1^{b,c}	3.2±0.2^{b,c}	3.4±0.3^{c}	2.9±0.0^{a,b}	2.5±0.2^{a}	3.0±0.2^{b}
1-octen-3-ol	13.7±1.3^{b}	20.6±0.1^{c}	20.7±0.1^{c}	21.5±3.1^{c}	19.7±0.2^{c}	4.3±0.6^{a}	23.5±2.2^{c}
1-heptanol	42.7±2.0^{a}	50.7±3.4^{a}	52.8±0.8^{a}	57.1±5.7^{a}	50.9±0.7^{a}	53.3±11.0^{a}	77.1±6.7^{b}
1-octanol	11.5±1.6^{a}	9.2±1.3^{a}	132±6^{c}	12.9±0.9^{a}	10.3±1.3^{a}	6.8±1.3^{a}	25.6±1.3^{b}
**Σ Alcohols**	**446±34**^{a}	**575±41**^{b,c,d}	**691±4**^{d}	**647±22**^{c,d}	**466±28**^{a,b}	**530±111**^{a,b,c}	**581±45**^{b,c,d}
butanoic acid	25.1±1.4^{a}	32.2±0.5^{a}	29.8±2.0^{a}	36.2±1.5^{a}	24.4±0.4^{a}	125±19^{b}	43.3±2.9^{a}
3-methylbutanoic acid,	838±68^{a}	1255±53^{b}	966±134^{a,b}	1043±74^{b}	851±35^{a}	1521±104^{d}	1023±14^{a,b}
hexanoic acid	1721±23^{b}	1722±50^{b}	1750±29^{b}	1786±89^{b}	1455±33^{a}	4031±240^{c}	1590±65^{a,b}
2-hexenoic acid	2051±22^{b}	297±36^{c}	236±9^{b,c}	252±52^{b,c}	296±20^{c}	61.2±15.3^{a}	221±27^{b}
2-methylpropanoic acid	114±16^{a,b}	72.5±2.7^{a}	142±12^{b}	164±23^{b}	116±6^{a,b}	147±46^{b}	136±32^{b}
octanoic acid	1343±127^{b,c}	1291±25^{b,c}	1490±9^{c}	1179±42^{b}	796±61^{a}	1328±262^{b,c}	864±76^{a}
**Σ Acids**	**4247±139**^{b,c}	**4670±61**^{c}	**4614±194**^{c}	**4461±155**^{c}	**3539±73**^{a}	**7213±490**^{d}	**3876±65**^{a,b}
ethyl butyrate	39.9±1.1^{a}	65.8±0.1^{b}	93.2±18.3^{c}	53.7±2.2^{a,b}	69.7±4.6^{b}	96.4±3.4^{c}	92.6±9.4^{c}
isoamyl acetate	1344±205^{c}	1041±69^{b}	1302±9^{c}	1545±111^{d}	1091±4^{b}	1650±36^{d}	1067±64^{b}
ethyl hexanoate	162±29^{a}	196±5^{b}	208±9^{b}	203±16^{b}	163±2^{a}	221±12^{b}	197±3^{b}
ethyl piruvate	18.9±2.1^{b}	40.8±0.7^{d}	25.8±6.8^{b,c}	28.6±5.9^{c}	26.0±1.5^{b,c}	8.2±2.2^{a}	16.3±5.0^{b}
hexyl acetate	16.7±1.9^{b}	17.7±0.8^{b}	24.7±3.7^{c}	14.9±4.6^{b}	9.0±1.7^{a}	4.8±0.8^{a}	19.6±0.8^{b}
ethyl lactacte	2137±518	2575±31	2589±142	2617±194	2026±95	2003±101	2633±357
ethyl 2-hydroxy-3-methylbutyrate	9.0±0.5^{a}	12.2±1.7^{a,b}	13.0±1.2^{b}	9.8±1.8^{a}	9.3±0.5^{a}	21.9±1.7^{c}	14.3±1.6^{b}
ethyl octanoate	190±25^{a}	193±13^{a}	148±7^{a}	186±32^{a}	131±2^{a}	352±63^{b}	116±2^{a}
ethyl 3-hydroxybutyrate	66.0±16.4^{a,b}	69.4±1.7^{a,b}	93.1±4.8^{b}	53.7±7.4^{a}	40.8±4.0^{a}	168±31^{c}	61.7±7.1^{a,b}
ethyl 2-hydroxy-4-methylpentanoate	32.9±6.1^{a,b}	40.8±7.2^{b,c}	32.7±0.8^{a,b}	24.6±4.8^{a}	29.5±1.2^{a,b}	48.8±2.0^{c}	34.3±9.6^{a,b}
ethyl decanoate	79.6±3.6^{c,d}	90.7±10.0^{d}	69.6±3.8^{c}	78.4±13.0^{c,d}	56.1±0.5^{b}	31.1±7.4^{a}	27.4±0.6^{a}

Supplementary Table 3. Continued.

ethyl succinate	456±44^{a}	428±6^{a}	420±11^{a}	394±50^{a}	410±23^{a}	954±95^{b}	351±17^{a}
1,3-propanediol diacetate	166±21^{a}	138±20^{a}	160±11^{a}	229±27^{a}	152±18^{a}	427±112^{b}	160±45^{a}
ethyl 4-hydroxybutyrate	968±165^{a}	1002±44^{a}	1002±37^{a}	1112±277^{a}	937±26^{a}	3848±311^{b}	1063±189^{a}
ethyl dodecanoate	3.2±0.3^{a}	5.8±1.4^{b}	8.0±0.3^{c}	3.2±0.0^{a}	5.2±0.2^{b}	2.5±0.1^{a}	5.7±0.3^{b}
diethyl malate	145±10^{a}	140±0^{a}	83.7±63.6^{a}	95.1±17.8^{a}	228±48^{b}	72.6±5.9^{a}	148±14^{a}
diethyl glutarate	156±26^{a}	150±10^{a}	157±21^{a}	115±17^{a}	253±16^{b}	345±67^{c}	190±51^{a}
monomethyl succinate	3742±216^{a}	5522±179^{b,c}	4718±566^{a,b}	4329±197^{a,b}	3567±152^{a}	6775±890^{c}	3111±1588^{a}
**Σ Esters**	**9733±701**^{a}	**11729±390**^{a}	**11150±684**^{a}	**11094±145**^{a}	**9202±119**	**15409±1371**^{b}	**9307±1811**^{a}
2-phenylethanol	27271±5959^{a}	58572±1795^{c}	55513±5095^{c}	46669±2086^{b,c}	34659±3216^{a,b}	39070±8822^{a,b}	53953±9868^{c}
benzenalcetaldehyde	610±28^{c}	570±10^{b,c}	577±20^{b,c}	641±51^{c}	574±18^{b,c}	490±70^{a}	434±50^{a,b}
benzoic acid	261±4^{a}	472±88^{a,b}	505±44^{a,b}	927±447^{b}	512±32^{a,b}	605±83^{a,b}	656±73^{a,b}
benzeneacetic acid	111±4^{a}	164±9^{b}	84.0±50.3^{a}	105±0^{a}	95.0±3.1^{a}	91.6±2.5^{a}	107±23^{a}
2-phenylethyl acetate	132±27^{b,c}	156±28^{c}	130±8^{b,c}	129±12^{b,c}	87.5±0.5^{a,b}	77.0±15.8^{a}	97.7±27.9^{a,b}
phenol	24.1±4.4^{a,b}	47.6±9.3^{d}	42.8±5.4^{c,d}	33.1±8.4^{,c}	50.6±0.9^{d}	17.9±3.3^{a}	54.0±9.2^{d}
acetovanillone	31.0±5.8^{a}	54.4±17.8^{b}	36.8±0.4^{a,b}	30.2±1.7^{a}	27.8±0.5^{a}	41.6±6.5^{a,b}	43.7±3.9^{a,b}
zingerone	11.0±1.0^{b}	13.3±0.9^{c}	14.5±0.4^{c}	14.5±1.2^{c}	12.5±0.1^{b,c}	5.4±0.6^{a}	17.5±1.2^{d}
vainillin methyl ether	46.1±6.9^{a,b}	38.6±8.9^{a}	48.6±0.0^{a,b}	51.8±8.8^{a,b}	59.0±3.6^{b}	36.2±3.7^{a}	148±9^{c}
**Σ Benzenic compounds**	**28497±5973**^{a}	**60090±1872**^{c}	**56954±5065**^{c}	**48603±1634**^{b,c}	**36079±3160**^{a,b}	**40436±8985**^{,b}	**55512±10066**^{c}
ethyl 3-(methylthio)-propionate	1.9±0.4^{b}	ND	ND	1.8±0.4^{b}	1.8±0.1^{b}	0.76±0.09^{a}	1.4±0.7^{b}
3-(methylthio)-1-propanol	397±33^{a,b}	293±9^{a,b}	321±20^{a,b}	376±74^{a,b}	264±16^{a}	439±118^{b}	295±67^{a,b}
2-mercaptoethanol	10.1±1.8^{a}	13.0±1.0^{a,b}	10.0±2.0^{a}	9.8±1.6^{a}	9.6±1.4^{a}	28.3±1.0^{c}	14.3±1.8^{b}
3-(methylthio)-propanoic acid	13.1±2.9^{b}	11.0±3.1^{b}	11.8±0.9^{b}	12.3±3.5^{b}	8.2±0.2^{a,b}	4.1±1.6^{a}	10.6±3.9^{b}
**Σ Sulphur compounds**	**422±35**^{b}	**317±5**^{a,b}	**343±23**^{a,b}	**400±76**^{b}	**284±18**^{a}	**404±4**^{b}	**322±74**^{a,b}
γ-butyrolactone	30.6±3.6^{a}	49.4±3.3^{a,b}	46.5±0.6^{a,b}	88.7±4.9^{b,c}	41.6±39.1^{a,b}	107.0±24.6^{c}	81.4±24.5^{b,c}
γ-nonanolactone	18.3±0.6^{a}	21.3±3.0^{a}	17.2±1.6^{a}	16.7±4.5^{a}	37.5±0.5^{b}	19.9±0.6^{a}	21.3±2.3^{a}
pantolactone	13.4±2.8	17.7±1.6	20.3±4.3	19.1±5.6	19.6±1.2	14.7±4.8	18.3±1.5
maltol	49.2±28.6^{b}	51.9±2.1^{b}	53.1±2.0^{b}	26.0±12.6^{a,b}	36.8±2.6^{b}	8.1±0.5^{a}	84.4±15.9^{c}
**Σ Furan & pyran compounds**	**112±28**^{a}	**141±10**^{a,b}	**137±5**^{a,b}	**150±3**^{a,b}	**176±2**^{b,c}	**150±23**^{a,b}	**205±41**^{c}

Values with different superscripts in the same row denoted significant differences according to the Student-Newman-Keuls test at $p < 0.05$. C-48: control wine with 48 h of skin maceration; C-72: control wine with 72 h of skin maceration; S20-48: wine from grape sonicated at 20 kHz with 48 h of skin maceration; S28-72: wine from grape sonicated at 28 kHz with 72 h of skin maceration; C-7d: control wine with 7 days of skin maceration. ND: not detected.
